# Synthetic and semi-synthetic approaches to unprotected *N*-glycan oxazolines

**DOI:** 10.3762/bjoc.14.30

**Published:** 2018-02-15

**Authors:** Antony J Fairbanks

**Affiliations:** 1 Department of Chemistry, University of Canterbury, Private Bag 4800, Christchurch, 8140, New Zealand; 2 Biomolecular Interaction Centre, University of Canterbury, Private Bag 4800, Christchurch 8140, New Zealand

**Keywords:** DMC, ENGase, glycosyl oxazolines, *N*-glycans, oligosaccharides

## Abstract

*N*-Glycan oxazolines have found widespread use as activated donor substrates for endo-β-*N*-acetylglucosaminidase (ENGase) enzymes, an important application that has correspondingly stimulated interest in their production, both by total synthesis and by semi-synthesis using oligosaccharides isolated from natural sources. Amongst the many synthetic approaches reported, the majority rely on the fabrication (either by total synthesis, or semi-synthesis from locust bean gum) of a key Manβ(1–4)GlcNAc disaccharide, which can then be elaborated at the 3- and 6-positions of the mannose unit using standard glycosylation chemistry. Early approaches subsequently relied on the Lewis acid catalysed conversion of peracetylated *N*-glycan oligosaccharides produced in this manner into their corresponding oxazolines, followed by global deprotection. However, a key breakthrough in the field has been the development by Shoda of 2-chloro-1,3-dimethylimidazolinium chloride (DMC), and related reagents, which can direct convert an oligosaccharide with a 2-acetamido sugar at the reducing terminus directly into the corresponding oxazoline in water. Therefore, oxazoline formation can now be achieved in water as the final step of any synthetic sequence, obviating the need for any further protecting group manipulations, and simplifying synthetic strategies. As an alternative to total synthesis, significant quantities of several structurally complicated *N*-glycans can be isolated from natural sources, such as egg yolks and soy bean flour. Enzymatic transformations of these materials, in concert with DMC-mediated oxazoline formation as a final step, allow access to a selection of *N*-glycan oxazoline structures both in larger quantities and in a more expedient fashion than is achievable by total synthesis.

## Review

### Introduction

Glycosyl oxazolines are high-energy intermediates on the hydrolytic pathway of some [1–5] (but not all) [6] of the numerous glycosidases that hydrolyse linkages between 2-acetamido sugars and other species. In particular the endo-β-*N*-acetylglucosaminidases [7] (ENGases, EC 3.2.1.96), a class of enzyme which specifically cleave between the innermost two GlcNAc residues of *N*-glycans attached to N-linked glycoproteins, all operate via a two-step mechanism involving neighbouring group participation of the 2-acetamide group and an oxazoline as a high energy intermediate [8].

Glycosyl oxazolines first drew the attention of synthetic chemists due to their use as glycosyl donors for the synthesis of oligosaccharides that comprise 2-amino-2-dexoy sugars [9]. Though the majority of synthetic work focussed on production and reaction of *gluco*-configured oxazolines (i.e., those derived from GlcNAc), the corresponding *manno* [10–11] and *galacto*-configured [12] compounds have also been made and studied. Although the first generation of these oxazoline donors [13–14] proved to be rather unreactive, and found only limited applications [15–18], the addition of three chlorines to the methyl group did increase their potency [19–23]. However, applications were still less widespread than more conventional glucosamine-derived donors.

**Scheme 1:**
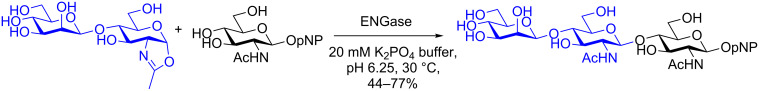
The first ENGase-catalysed glycosylation of a GlcNAc acceptor using an *N*-glycan oxazoline as donor.

Resurgent interest in the production of glycosyl oxazolines, and in particular oxazoline derivatives of *N*-glycans, was as a direct result of their utility as activated donors species for glycosidase-catalysed synthesis [24–27]. Initially activity centred on the use of oxazolines as donors for chitinase-catalysed glycosylations [28–31]. However, a turning point occurred when, in a seminal publication in 2001 Shoda [32] and co-workers reported that a disaccharide oxazoline (Scheme 1) was an effective donor substrate for two ENGase enzymes (Endo A and Endo M), both of which were capable of using it to glycosylate two GlcNAc acceptors, to produce trisaccharide products.

Subsequently the ENGases in combination with *N*-glycan oxazolines, have become the biocatalysts of choice for the convergent production of a wide variety of biologically interesting glycopeptides and for the remodelling of glycoproteins, including mAbs [33–34]. The efficient production of *N*-glycan oxazolines as donor substrates for these enzymes has therefore become an area of significant interest over the past 15 years [35–36].

### The synthesis of *N*-glycan oxazolines

#### Formation of glycosyl oxazolines

**Scheme 2:**
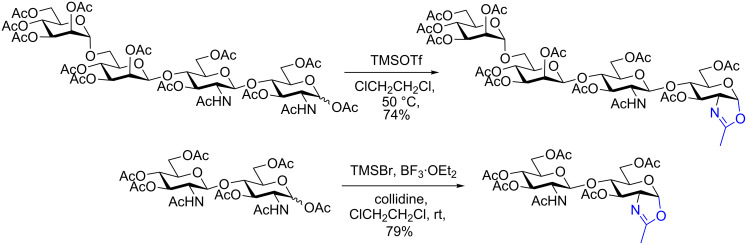
Production of *N*-glycan oxazolines from peracetylated sugars using Lewis acids.

Glycosyl oxazolines of monosaccharides can be produced straightforwardly using strong Lewis acids (e.g., FeCl_3_, SnCl_4_, or TMSOTf) and a fully protected (typically peracetylated) GlcNAc or other 2-acetamido sugar [37–40]. Oxazoline formation is achieved by activation of the leaving group at the anomeric centre and neighbouring group participation by the 2-acetamide. Unfortunately application of these reaction conditions to oligosaccharide substrates leads to significant cleavage of interglycosidic linkages, and correspondingly low yields of products. However, two methods that are useful for the production of oligosaccharide oxazolines are treatment of the peracetylated sugar with either TMSOTf in dichloroethane [39], or with TMSBr, BF_3_·Et_2_O and 2,4,6-collidine in dichloroethane [40] (Scheme 2). Both procedures give oxazolines of *N*-glycans in moderate to good yield with no cleavage of the oligosaccharide chain; the latter method reportedly gives better yields of more structurally complex *N*-glycan oxazolines.

However, employing protected sugars as substrates presents some limitations, as any remaining protecting groups must be removed in a subsequent step. Firstly, and most importantly, glycosyl oxazolines are extremely labile to acidic hydrolysis, and so this approach precludes the use of any OH-protecting groups that require acidic conditions for their cleavage. Secondly some glycosyl oxazolines are also prone to reductive cleavage by catalytic hydrogenation [41], presenting a significant further limitation as to which OH-protecting groups may be employed. Most of the reports in the literature have therefore used a protecting group regime in which all of the sugar hydroxy groups have been protected with base-labile groups, most commonly acetate esters. Importantly glycosyl oxazolines are completely stable to the typical basic conditions used for ester removal (e.g., Zemplen deacetylation). The generally accepted approach (until 2009) was therefore to perform all protecting group manipulations/interconversions on the completed oligosaccharide to ensure that all OH groups were protected as base-labile esters, before oxazoline formation.

**Scheme 3:**
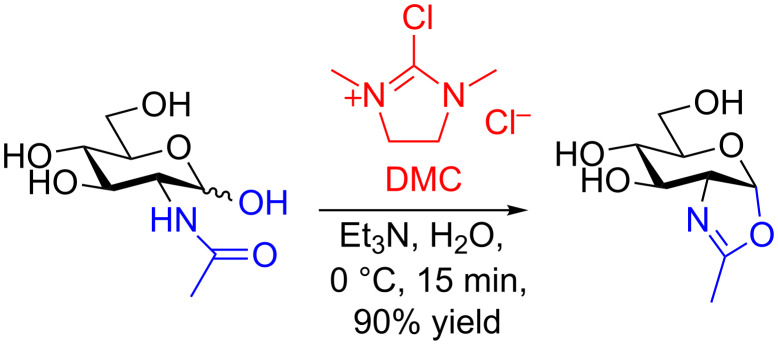
Direct conversion of unprotected GlcNAc to a glycosyl oxazoline by treatment with DMC and Et_3_N in water.

In 2009, Shoda published [42] a paper that was to completely change the way in which glycosyl oxazolines were made, and which would ultimately make many more readily available. In this seminal work, Shoda reported that the treatment of GlcNAc in aqueous solution with the activating agent 2-chloro-1,3-dimethylimidazolinium chloride (DMC) in the presence of triethylamine as the base, led to the formation of the glycosyl oxazoline in good yield (Scheme 3). Moreover this remarkable transformation was equally applicable to considerably larger oligosaccharide structures (vide infra). This breakthrough changed the way that all unprotected *N*-glycan oxazolines were to be made from that point in time onwards.

Although in later papers Shoda has published alternative reagents that may be used to achieve the same transformation, such as 2-chloro-1,3-dimethyl-1*H*-benzimidazol-3-ium chloride (CDMBI) [43], DMC remains the most popular reagent for glycosyl oxazoline production. DMC is remarkably tolerant of other functional groups in the oligosaccharide, for example sialic acids [44] and phosphates [45–46] are completely unaffected; the former is perhaps rather surprising since DMC was first developed as a carboxylic acid activating agent for peptide synthesis by Ishikawa [47]! One caveat to the procedure is that it is considerably less efficient for GalNAc; in this case the corresponding oxazoline is only produced in ≈50% yield. Indeed some of the other very useful DMC-mediated transformations of unprotected reducing sugars in aqueous solution that have been developed recently also work less effectively when the sugar at the reducing terminus has a *galacto* configuration [48–50].

#### Production of unprotected *N*-glycan oxazolines by total synthesis

**Scheme 4:**
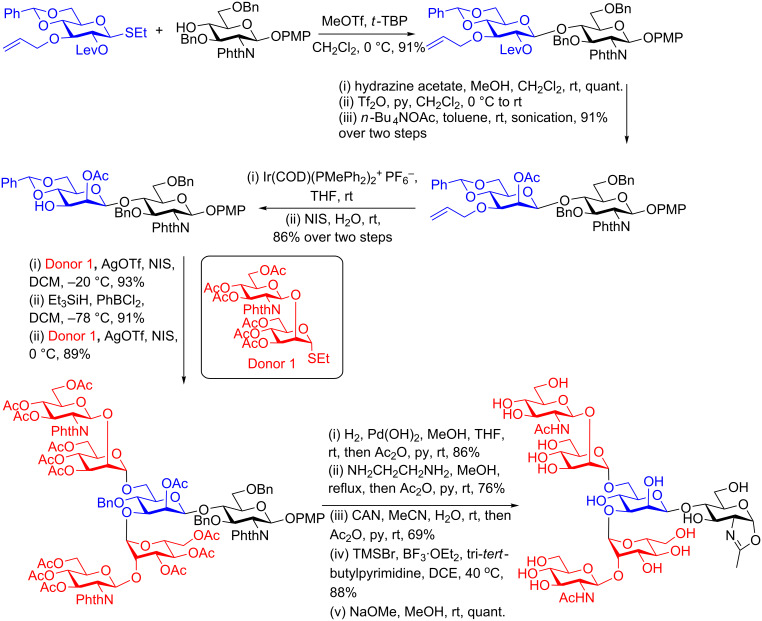
Total synthesis of a truncated complex biantennary *N*-glycan oxazoline via an epimerisation approach and Lewis acid mediated oxazoline formation.

The majority of the reported syntheses of *N*-glycan oxazolines have employed a key selectively protected Manβ(1–4)GlcNAc disaccharide building block which has then been extended at the 3- and 6-positions of the branching mannose unit. Amongst the possible ways to synthesise this key disaccharide [51–52] two have been used predominantly for the synthesis of *N*-glycan oxazolines. The OH-2 epimerisation approach, which uses a *gluco-*configured donor for glycosylation of the OH-4 of a selectively protected glucosamine acceptor has been used more than the other methods. Selective and orthogonal protection of OH-2 of the donor by an ester group facilitates both the stereoselective formation of the desired β-linkage, and also access to OH-2 after glycosylation for epimerisation. Amongst the many syntheses [53–58] of *N*-glycan oxazolines using this approach, the use of Lev protection on the donor, first developed by Boons [59], and then triflation and nucleophilic substitution by acetate aided by sonication, first developed by Fürstner [60–61], appear to be optimal. An example that employed these key steps was used to synthesise a truncated complex biantennary *N*-glycan oxazoline [62], as shown in Scheme 4. Following the *gluco* to *manno* epimerisation process, selective deprotection of OH-3 of the mannose unit was followed by glycosylation and extension of the 3-branched arm. Subsequent removal (or regioselective reductive ring-opening) of the 4,6-benzylidene protecting group allowed a second glycosylation at position 6. Conversion of all OH-protecting groups to acetate and the phthalamide to acetamide was followed by oxazoline formation using TMSBr, BF_3_·Et_2_O and 2,4,6-collidine in dichloroethane, and finally deacetylation. Modifications of this basic strategy have allowed the synthesis of a wide variety of truncated and structurally modified glycans [53–59].

**Scheme 5:**
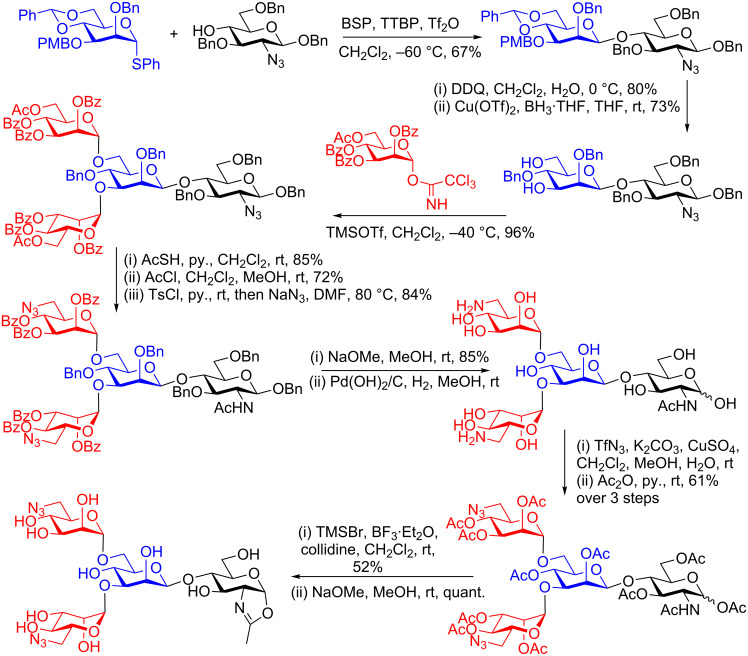
Wangs’s total synthesis of an *N*-glycan oxazoline incorporating click handles, employing Crich direct β-mannosylation.

Amongst other synthetic approaches that may be used to access the ‘difficult’ Manβ(1–4)GlcNAc linkage, including a variety of methods of intramolecular glycosylation [63–71] the most widely applied has been the Crich direct β-mannosylation [72–76]. However, one apparent limitation is that generally the reaction only works well if the GlcNAc acceptor has an azide or sulfonamide at position 2, rather than acetamide or *N*-phthalamide. Scheme 5 shows an example of the synthesis of a modified core *N*-glycan tetrasaccharide oxazoline from the several reported by Wang [77] using this approach. In this case following formation of the key Manβ(1–4)GlcNAc disaccharide both the 3- and 6-hydroxy groups of the mannose residue were deprotected, and the resulting diol underwent a double glycosylation with a selectively protected trichloroacetimidate donor.

An added advantaged of approaches that use total synthesis is the possibility of the incorporation of tags into the glycan structure, which allows further modifications to be made later. In this case, following conversion of the azide at position 2 of the glucosamine unit into an acetamide, azide was introduced at position 6 of the two terminal mannose residues. Protecting group interconversions, and peracetylation were followed by conversion to the oxazoline, using TMSBr, BF_3_·Et_2_O and collidine, and finally deacetylation. It was found that the incorporated azide was tolerated by the ENGase enzyme (Endo A), and so a modified glycoprotein (RNase) was made by enzymatic attachment of this synthetic tetrasaccharide, to which other species were then conjugated by click reactions.

**Scheme 6:**
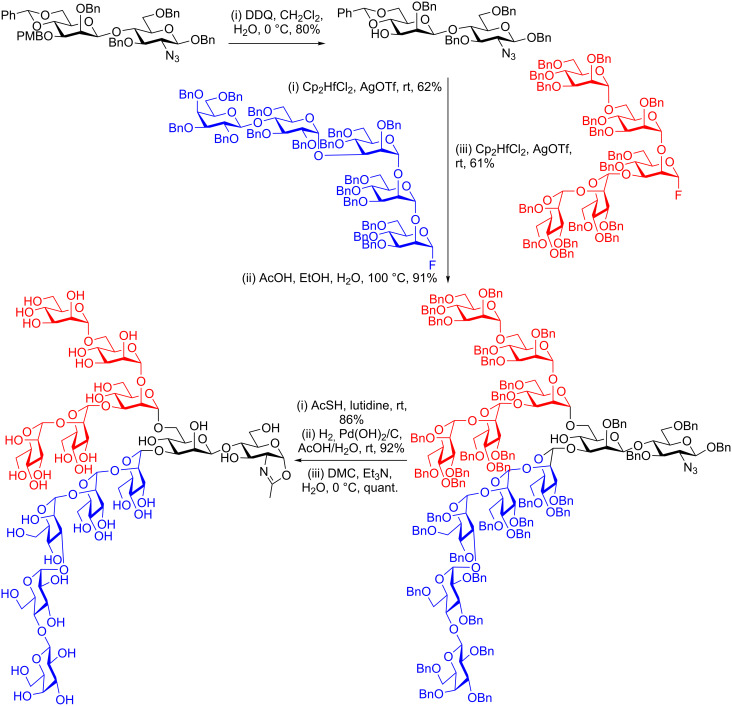
Wangs’s total synthesis of an *N*-glycan dodecasaccharide oxazoline employing final step oxazoline formation with DMC.

In more recent examples conversion of the completely deprotected glycan to the oxazoline by treatment with DMC has become the normal (and most effective) strategy. For example the same key Manβ(1–4)GlcNAc disaccharide was used by Wang for the more extended synthesis of a dodecasaccharide oxazoline (Scheme 6) [78]. In this case selective removal of the PMB protecting group at OH-3 was followed by glycosylation with a pentasaccharide glycosyl fluoride donor, comprising one galactose, one glucose, and three mannose residues. Acid catalysed hydrolysis of the 4,6-benzylidene was followed by regioselective glycosylation of the primary 6-OH with a different pentasaccharide, this time comprised of five mannoses. Conversion of the azide to acetamide and removal of all benzyl groups by hydrogenolysis produced a completely deprotected dodecasaccharide, which was finally converted to the glycosyl oxazoline by treatment with DMC in quantitative yield.

**Scheme 7:**
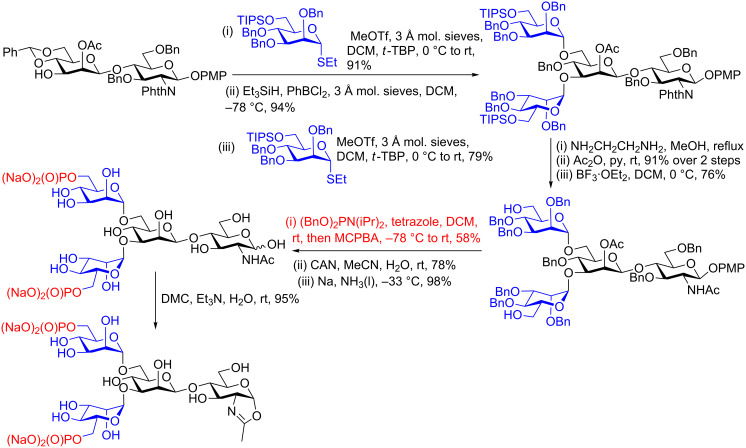
Production of a phosphorylated *N*-glycan oxazoline, employing final step oxazoline formation with DMC.

Final stage conversion of the fully deprotected oligosaccharide into the oxazoline has greatly facilitated the synthesis of more complex *N*-glycan oxazolines by analogous routes [79–80], including those bearing mannose-6-phosphate residues [45–46]. For example as shown in Scheme 7 sequential glycosylation of the key Manβ(1–4)GlcNAc disaccharide at positions 3 and 6, using the same selectively protected *manno* thioglycoside donor gave a tetrasaccharide. Removal of the silyl protecting groups revealed the 6-hydroxy groups of the terminal mannose residues, which were then phosphorylated. Removal of the anomeric PMP protection was followed by global deprotection by Birch reduction to give the completely deprotected tetrasaccharide diphosphate. Finally treatment with DMC in water in the presence of Et_3_N resulted in conversion to the glycosyl oxazoline in an excellent 95% yield.

#### Semi-synthesis: the locust bean gum approach

**Scheme 8:**
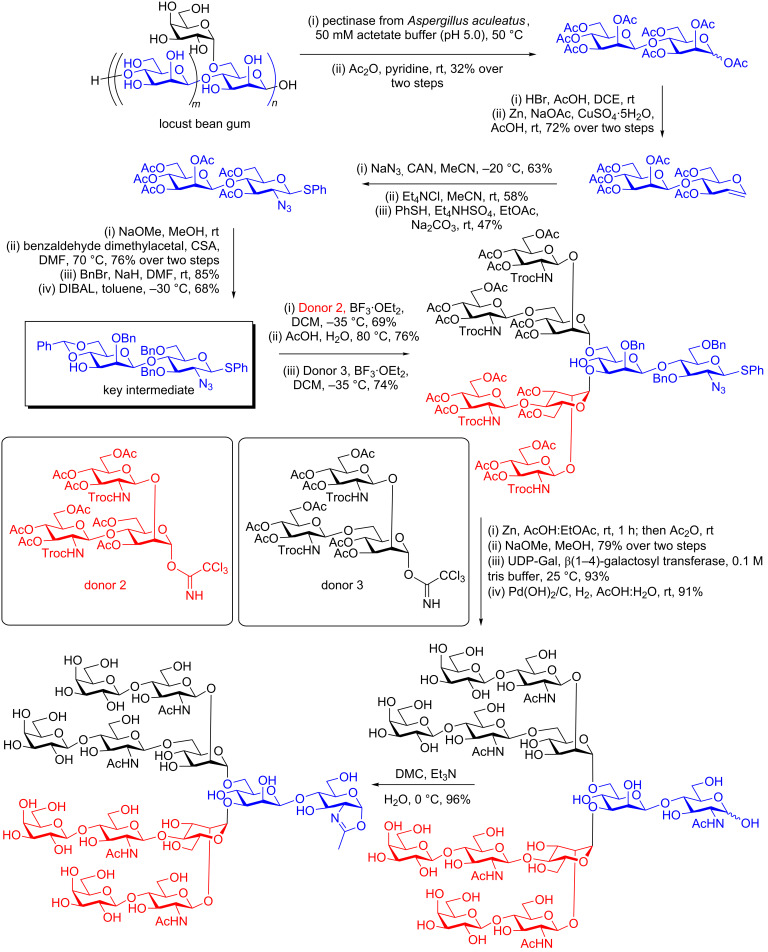
Enzymatic degradation of locust bean gum, and chemical conversion into an *N*-glycan dodecasaccharide oxazoline.

The naturally occurring polysaccharide locust bean gum contains a repeating Manβ(1–4)Man disaccharide unit, which is also decorated with branching α-galactose residues attached to OH-6 of some of the mannoses. Nishimura and co-workers [81] realised the potential utility of this Manβ(1–4)Man disaccharide in an expedient route to the part of *N*-glycans that is most difficult to synthesise; namely the Manβ(1–4)GlcNAc linkage. Treatment of locust bean gum with pectinase from *Aspergillus aculeatus*, which has both mannosidase and galactosidase activity, at 50 °C for 48 h in a 50 mM acetate buffer (pH 5.0) resulted in the production of a mixture of compounds, from which the Manβ(1–4)Man disaccharide was readily purified by acetylation (typically in ≈30% overall yield, Scheme 8). In the key transformation, the mannose residue at the reducing terminus was then converted into a glucosamine derivative (in fact possessing an azide at C2) first by conversion to the glycal and then an azido nitration reaction. This innovative method is considerably shorter than other approaches to the Manβ(1–4)GlcNAc (or equivalent) disaccharide. Elegant protecting group manipulations, involving the formation of a dibenzylidene derivative on the mannose ring, benzylation of the remaining free hydroxy groups on the glucosamine ring, and then regio- and chemoselective reductive ring opening of the less stable 5-ring benzylidene with DIBAL, led to a key disaccharide intermediate in which OH-3 of the mannose unit was unprotected and in which the 4- and 6-positions were protected as a benzylidene. Extension of this core disaccharide should be straightforward by traditional synthetic methodology, and so in principle the locust bean gum approach should allow rapid access to a wide variety of more extended *N*-glycan structures. In their original publication Nishimura and co-workers first glycosylated the free OH at position 3 with 2,4-branched trisaccharide trichloroacetimidate donor 2, removed the 4,6-benzylidene, and then regioselectively glycosylated the free primary OH at position 6 with 2,6-branched trisaccharide trichloroacetimidate donor 3. Following conversion of the Troc groups into acetamides and reduction and acetylation of the azide, all of the acetates were removed. Treatment with UDP-Gal and a β(1–4)-galactosyl transferase led to the addition of galactose residues to all of the 4-hydroxy groups of the GlcNAcs. Deprotection of the remaining benzyl protecting groups and removal of the SPh at the reducing terminus by catalytic hydrogenation gave the completely deprotected dodecasaccharide. Finally conversion to the corresponding oxazoline by the use of DMC gave the tetraantennary complex *N*-glycan oxazoline in 96% yield (Scheme 8).

### Production of *N*-glycan oxazolines using oligosaccharides isolated from natural sources

#### The egg yolk approach

The yolk of hens’ eggs contains a glycopeptide, often termed sialylglycopeptide (SGP), which is comprised of a short peptide linked to a complex biantennary *N*-glycan. Thus egg yolks can serve as a source of this complex biantennary *N*-glycan [(NeuAcGalGlcNAcMan)_2_ManGlcNAc_2_], following isolation of SGP, and subsequent enzymatic degradation. The original procedure [82] for the isolation of SGP first involved deproteinization by treatment with 90% phenol and washing with Et_2_O, and then repeated purification by size exclusion chromatography (SEC, Sephadex G-50, followed by Sephadex G-25) from which sialic acid positive fractions were collected. Further purification by anion exchange chromatography (Sephadex DEAE eluting with NaCl) removed any non-sialylated glycans, and was followed by cation exchange chromatography (Sephadex C-25). Finally desalination using SEC (Sephadex G-25) gave pure SGP.

**Scheme 9:**
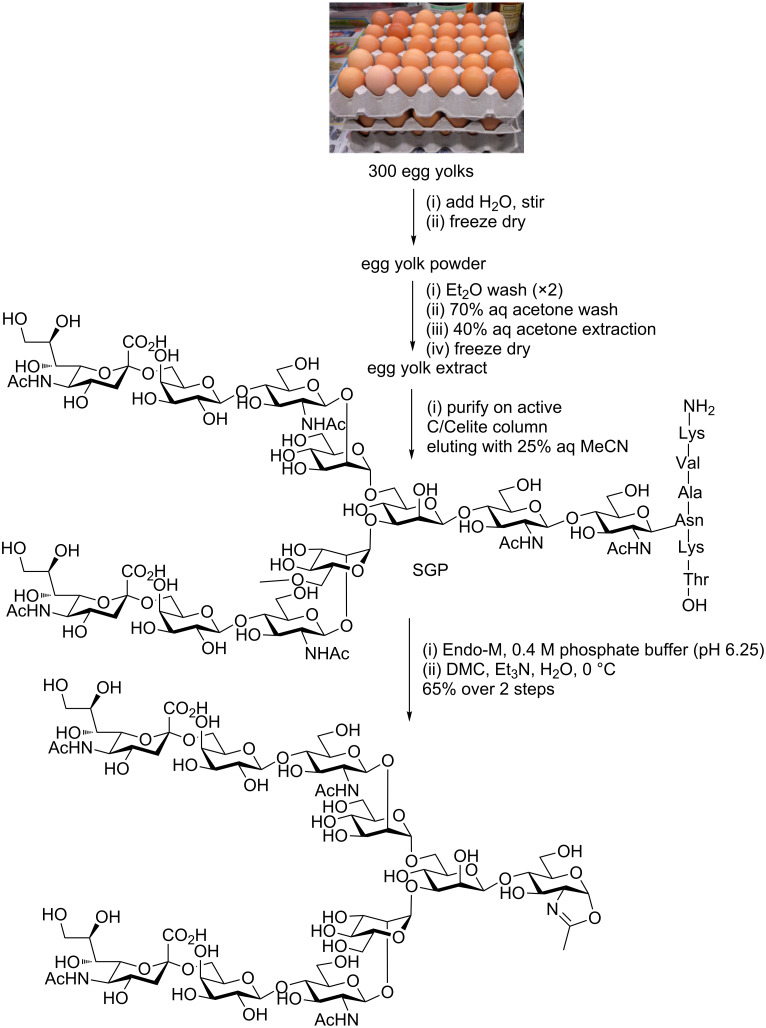
Production of a complex biantennary *N*-glycan oxazoline from hens’ eggs by semi-synthesis via isolation of SGP, enzymatic degradation, and final stage oxazoline formation.

Several improvements have subsequently been published which have made the isolation process easier and improved the yield. Firstly a significantly shortened procedure [83] followed the phenol treatment with a single purification by SEC (Sephadex G-50), and then filtration through graphitized carbon cartridges. Subsequently an even better method was developed [84] which avoided the treatment with phenol and all SEC purification steps (Scheme 9). In this process the egg yolks were first stirred with water and then freeze dried to give egg yolk powder. This powder was washed successively with diethyl ether and then 70% aqueous acetone. The solid was then extracted by vigorous mixing with 40% aqueous acetone. Following filtration through Celite^®^, the filtrate was concentrated and freeze-dried. The powder was dissolved in water and then purified on an active carbon/Celite^®^ (2:1) column, eluting with 25% MeCN, to give pure SGP on a gram scale; typically 1.5–2.0 g of SGP is obtained from 300 eggs.

Very recently Boons and co-workers [85] published further modifications and optimisation of this procedure, and reported that it is possible to start with commercially produced lyophilised egg yolk powder, rather than the eggs themselves. Their method, which also included purification by the use of preparative hydrophilic interaction chromatography–high performance liquid chromatography (HILIC–HPLC), clearly reduces time and effort by removing the need for separation of the yolks and freeze-drying. However, care has to be exercised with respect to the processing that the commercially sourced egg yolk powder has undergone; for example spray drying at >100 °C may lead to degradation of the glycans.

Whichever method of SGP production is used, the free oligosaccharide [(NeuAcGalGlcNAcMan)_2_ManGlcNAc] can then be released from SGP by treatment with the ENGase Endo M [86]. Following purification by SEC (Sephadex G-25), the free glycan can be converted into the oxazoline by treatment with DMC in water (Scheme 9), as first reported by Wang [87] and Umekawa and co-workers [88], and then subsequently used by others [44,89–90]. Shoda’s modified version of DMC (2-chloro-1,3-dimethyl-1*H*-benzimidazol-3-ium chloride, CDMBI) has also been reported to be efficient at this transformation [91]. Furthermore removal of the terminal sialic acid residues of the free oligosaccharide by treatment with a neuraminidase allows the production of truncated complex biantennary glycans. Originally Wang and co-workers reported [92] the synthesis of this type of oxazoline using a sequence of acetylation, treatment with TMSBr/BF_3_·Et_2_O/collidine and deacetylation. However, treatment of the free reducing sugar with DMC allows the production of the truncated complex *N*-glycan oxazoline in a more efficient manner [93].

In related work, Kajihara has recently developed [94] methods that allow selective modification of the complex biantennary *N*-glycan available from egg yolks. For example, after the peptide is degraded to a single Asn residue by protease digestion (Actinase E), the sialic acids can be removed by acidic hydrolysis [95], and the amine Fmoc protected. Branch specific exo-glycosidase digestion then allows the production of a wide variety of truncated glycans. Alternatively, by forming 4,6-benzylidenes of the mannose and galactose residues, acetylating all the remaining free OH groups, and then using mild acidic hydrolysis (60% aqueous acetic acid), Kajihara was able to produce a mixture of products in which either one or both of the mannose residues had been deprotected but the galactose residues remained completely protected. HPLC separation then allowed either selective chemical glycosylation or protection of the primary OH groups; the remaining secondary hydroxy group of the products of the latter process could also be glycosylated. Ultimately this methodology allows the synthesis of the considerably more complex *N*-glycans, for example tri- (and presumably in the future tetra-) antennary glycans, starting from SGP. Although the protecting group-based reactions lack complete selectivity and the sequences require several careful HPLC separations, the fact that the complex biantennary glycan is so readily available still makes these approaches attractive with respect to total synthesis. Neither Kajihara nor others have yet to employ these routes to the production of *N*-glycan oxazolines.

#### The soy bean approach

**Scheme 10:**
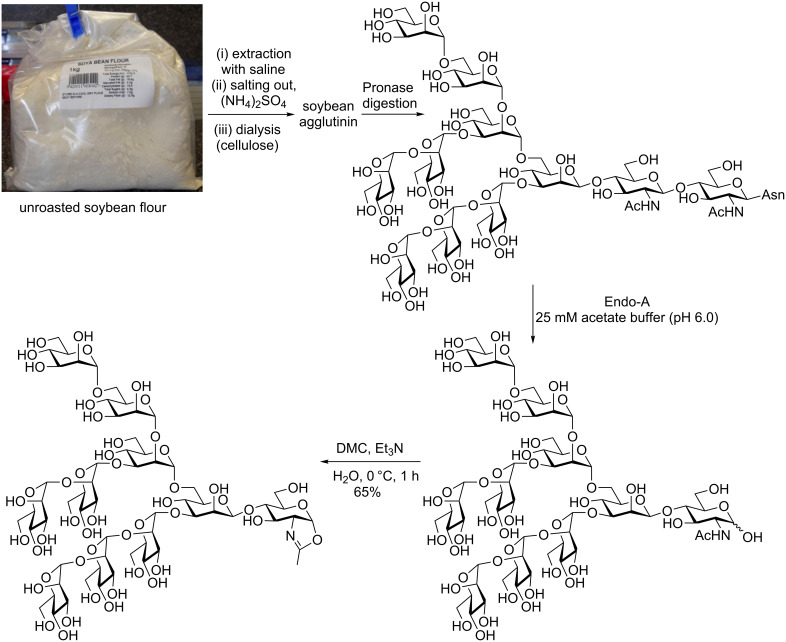
Production of a high mannose (Man-9) *N*-glycan oxazoline from soy bean flour.

Soy bean agglutinin is a glycoprotein decorated with high mannose glycans [96]. Isolation of soy bean agglutinin from unroasted soy bean flour is achieved by acidification (pH 4.6), and salting out with ammonium sulphate [97–98]. The Asn-linked Man-9 glycan (Man_9_GlcNAc_2_Asn) can then be prepared [99] by exhaustive Pronase digestion, followed by SEC (Sephadex G-50) and further purification with HPLC on a graphitized carbon column (Scheme 10) [100]. Alternatively the glycan may also be released by hydrazinolysis [101]. Following isolation of the full-length (Man_9_GlcNAc_2_) Asn-linked glycan the truncated glycan (Man_9_GlcNAc) can be produced by treatment with the ENGase Endo A [102], and purification by SEC (Sephadex G-15). The first production of the Man-9 oxazoline reported by Wang [103] then involved complete acetylation of this decasaccharide, treatment with TMSBr/BF_3_·Et_2_O/collidine, and a final deacetylation step. However, this method can now be simplified by use of the Shoda DMC procedure by which the unprotected Man_9_GlcNAc glycan can be directly converted to the oxazoline in water [104]. This route, although lower yielding than the corresponding egg yolk procedure, is still considerably more efficient than using total synthesis to make such a highly complex decasaccharide.

### Other routes to *N*-glycan oxazolines

**Scheme 11:**
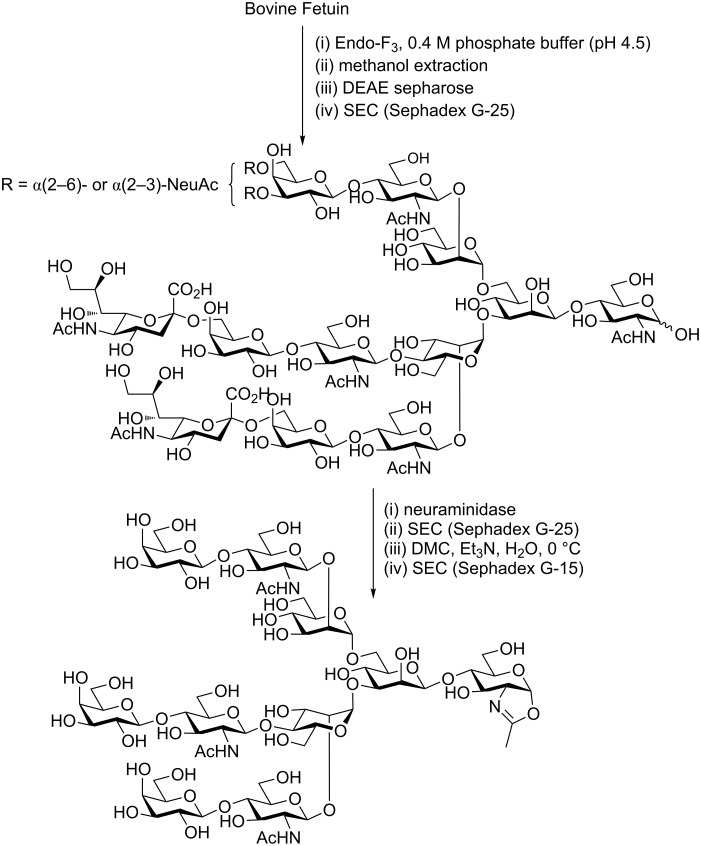
Production of a triantennary *N*-glycan oxazoline from bovine feruin by semi-synthesis.

Recently Wang and co-workers have reported [105] a semi-synthetic route to triantennary *N*-glycan oxazolines starting from bovine fetuin (Scheme 11). To enable large-scale production they first purified bovine fetuin from fetal bovine serum [106]. The *N*-glycans were then released by treatment with the ENGase Endo-F3 [107], and were partially purified by acetone precipitation and extraction with 60% methanol. The crude *N*-glycans were found to be a rather complex mixture of compounds, the four major components of which were identified as triantennary glycans with 2 or 3 sialic acids attached as regioisomers (i.e., both α(2–6)- and α(2–3)-linked to the 6-branched mannose arm). Isolation of these major compounds and treatment with a neuraminidase then produced essentially a single product, which was purified by SEC (Sephadex G-25), and finally converted to the corresponding triantennary oxazoline by treatment with DMC in water. Final purification was achieved by SEC (Sephadex G-15).

Commercially available chicken ovalbumin can be used as a source of high mannose *N*-glycans, from which glycopeptides can be obtained by pronase digestion [108]. A mixture of free truncated glycans (Man_5_GlcNAc and Man_6_GlcNAc) can then be released [109] by the use of the ENGase Endo A, and separated by careful chromatography on a carbon-Celite^®^ column. Although Wang and co-workers did not at that time report the conversion of these glycans into the corresponding oxazolines, the basic Man_5_GlcNAc structure has since been extended using a sequence of glycosyl transferases (namely a β(1–2)-GlcNAc transferase, a β(1–4)-galactosyltransferase, and an α(2–6)-sialyltransferase), and the corresponding hybrid *N*-glycan oxazoline used as a substrate for ENGases [110].

## Conclusion

*N*-Glycan oxazolines have found widespread use as activated donor substrates for ENGase enzymes, a factor which has in turn stimulated interest in their production both by total synthesis and semi-synthesis. By far the most significant recent breakthrough in the field has been the development by Shoda of DMC (and related reagents), which can effect the direct conversion of oligosaccharides with a 2-acetamido sugar at the reducing terminus directly into the corresponding glycosyl oxazoline in water. This ‘game-changer’ means that nowadays no protecting group manipulations are required after oxazoline formation, which is performed as the final step; this makes production by total synthesis considerably easier. Additionally the remarkable ability of DMC and related reagents to achieve this key transformation also facilitates the use of naturally derived oligosaccharides as useful sources of *N*-glycan oxazolines. Recent work has both simplified the isolation of such *N*-glycans from natural sources, such as egg yolks, and also extended the variety of structures available by such means. It seems likely that more *N*-glycans will become available by such methods in the future. Furthermore it also appears to be only a matter of time until homogeneous glycoproteins and other glycoconjugates produced using *N*-glycans oxazolines find therapeutic and other applications; a development which will further stimulate the search for even better methods for their large scale and cost-effective production.
